# Supplementary material for: Asynchronous magnetic resonance elastography: Shear wave speed reconstruction using noise correlation of incoherent waves
Source: Magn Reson Med. 2022 Oct 27;89(3):990–1001. doi: 10.1002/mrm.29502 (PMC9792433; doi:10.1002/mrm.29502)
Supplement: Supplementary file 1 — DATA S1 MATLAB code used to generate the numerical simulations of Figure 1 [file MRM-89-990-s002.zip › k-Wave/helpfiles/sharpness.html]

sharpness :: Functions (k-Wave)


# sharpness

Calculate image sharpness metric.

## Syntax

```
s = sharpness(im)
s = sharpness(im, metric)
```

## Description

`sharpness` returns a scalar metric related to the sharpness of the 2D or 3D image matrix defined by `im`. By default, the metric is based on the Brenner gradient which returns the sum of the centered finite-difference at each matrix element in each Cartesian direction. Metrics calculated using the Sobel operator or the normalised variance can also be returned by setting the input paramater `metric`.

For further details, see B. E. Treeby, T. K. Varslot, E. Z. Zhang, J. G. Laufer, and P. C. Beard, "Automatic sound speed selection in photoacoustic image reconstruction using an autofocus approach," J. Biomed. Opt., vol. 16, no. 9, p. 090501, 2011.

## Inputs

|  |  |
| --- | --- |
| `im` | 2D or 3D image data to evaluate |

## Optional Inputs

|  |  |
| --- | --- |
| `metric` | sharpness metric. Supported values are  `'Brenner'` (default)  `'Tenenbaum'`  `'NormVariance'` |

## Outputs

|  |  |
| --- | --- |
| `s` | computed sharpness metric |
